# Risk factors and nomogram predictive model of surgical site infection in closed pilon fractures

**DOI:** 10.1186/s13018-023-04058-z

**Published:** 2023-08-08

**Authors:** Chenrong Ke, Xiaoyu Dong, Guangheng Xiang, Juanjuan Zhu

**Affiliations:** 1 Department of Orthopaedic, The Second Affiliated Hospital and Yuying Children’s Hospital of Wenzhou Medical University, Wenzhou, 325035 Zhejiang China; 2 School of Pharmaceutical Sciences, Wenzhou Medical University, Wenzhou, 325035 Zhejiang China; 3 Department of Geriatrics and Neurology, The Second Affiliated Hospital and Yuying Children’s Hospital of Wenzhou Medical University, Wenzhou, 325035 Zhejiang China

**Keywords:** Pilon fracture, Surgical site infection, Risk factors, Nomogram

## Abstract

**Objectives:**

In this study, we try to investigate the risk factors of postoperative surgical site infection (SSI) in closed pilon fractures and establish a nomogram prediction model.

**Methods:**

From January 2012 to June 2021, 516 closed pilon fracture patients were included in this study. Of these, 387 patients were randomly assigned to the training group and 129 patients were assigned to the validation group (3:1). By univariate and multivariate Cox analysis, we identified independent risk factors for postoperative SSI after Pilon fracture. We established a nomogram model and used receiver operating characteristic (ROC) and calibration chart to evaluate its discriminant and calibration.

**Results:**

SSI occurred in 71 patients in the training group and 23 patients in the validation group. Ultimately, age, preoperative blood sugar, operative time, Tscherne classification and fracture classification were identified as independent risk factors for SSI. The AUC values for SSI of the training and validation group were 0.898 and 0.880, and the P value of the Hosmer–Lemeshow test was 0.125. We established a nomogram prediction model based on age, preoperative blood sugar, operative time, Tscherne classification and fracture classification.

**Conclusion:**

Our nomogram model had good discrimination and calibration power, so it could be used to predict SSI risk in patients with pilon fracture.

## Introduction

In 1911, French radiologist Etienne Destot first described pilon fractures as injuries to the distal tibia's articular weight-bearing surface [1]. The tibia pilon fracture makes up approximately 1% of all lower-extremity fractures and 5% to 10% of all tibia fractures, usually associated with severe soft tissue injury [2–4]. Pilon fractures are usually result from high-energy trauma and axial violence, such as skiing, car accidents, falls from great heights and so on [5, 6].

In the AO/OTA classification of long bone fractures, pilon fractures are classified as extra-articular (43A), partially articular (43B), and intra-articular (43C), and are further subdivided according to the degree of comminution [7]. For closed fractures, the degree of soft tissue injury is evaluated using the Tscherne classification [8]. The treatment of pilon fractures is dominated by surgery, and despite some progress, it remains challenging. Common complications after surgery include wound dehiscence, infection, nonunion, malunion, joint stiffness and post-traumatic arthritis [9–12].

Postoperative infection is often catastrophic, and there is even a risk of amputation [13]. Various authors have reported that infection rates after pilon fractures surgery ranging from 8.9 to 26.7% [14–16]. At present, smoking, diabetes, operation time, and open injury have been identified as potential risk factors for postoperative infection after ankle fracture, but the research on the risk factors for postoperative infection after closed pilon fractures is limited [17, 18].

In this study, we try to investigate the risk factors for postoperative surgical site infection (SSI) in closed pilon fractures and establish a nomogram prediction model. To provide a reference for the prevention and treatment of high-risk infection patients in the future.

## Materials and methods

### Inclusion and exclusion criteria

This study was approved by the Ethics Committee of our Institute (NO. 2021-K-241-01) in accordance with the guiding principles of the Declaration of Helsinki. All electronic medical records and image data were anonymised and personal identifiers were completely removed.

Patients who underwent surgical treatment for pilon fractures in our hospital from January 2012 to June 2021 were included in this retrospective study. The inclusion criteria were: (1) age ≥ 18 years; (2) the Arbeitsgemeinschaft für Osteosynthesefragen/Orthopaedic Trauma Association (AO/OTA) 43 pilon fracture; (3) closed fracture; (4) underwent open reduction and internal fixation (ORIF); (5) complete clinical data. Exclusion criteria were as follows: (1) open fracture; (2) pathological fracture; (3) tibia shaft fracture; (4) trimalleolar ankle fracture; (5) conservative treatment; (6) kirschner wire or external fixation. Finally, a total of 516 pilon fracture patients were enrolled in our study.

### Risk factors and outcome measures

Demographic information including, age, gender, hemoglobin, serum albumin, c-reactive protein (CRP), blood platelet, leukocyte, preoperative blood sugar, waiting time for surgery, current smoking status and drinking status were extracted from the medical records. Among the causes of injury were falling from height, traffic accident, hit by heavy object and other. Polytrauma was defined as trauma to more than one of the following systems: musculoskeletal, abdominal, cardiothoracic, urogenital, vascular, and central nervous systems. Fractures were classified as extra-articular (43A), partially articular (43B), and intra-articular (43C) according to the AO/OTA system [7]. The degree of soft tissue injury was assessed using the Tscherne classification: Grade 0 represents minimal tissue damage associated with simple fracture pattern; Grade 1 involves superficial abrasion or contusion; Grade 2 involves deep abrasion of skin or muscle contusion; Grade 3 presents with extensive skin and muscle damage or crush injury, subcutaneous avulsion, and/or compartment syndrome [8]. Where there was conflict in classification, group discussion was used to reach consensus. Factors related to surgery were also assessed, including operative time, intraoperative blood loss, surgical approach, bone graft, drainage and number of people in the operating room.

A staged approach was used for pilon fractures with severe soft tissue damage, first with external fixation of the tibia and/or restoration of fibula length, and then with delayed tibial open reduction and internal fixation after soft tissue improvement. We defined surgical site infection as any infection that occured at the surgical incision site or deep tissue within 30 days of surgery (within one year of implant used) according to the U.S. Centers for Disease Control and Prevention (CDC) [19]. SSI including superficial and deep infection, with or without positive cultures. The surgeon decided to use antibiotics, wound treatment and surgical treatment based on patient clinical symptom and wound condition.

### Statistical analysis

Patients were randomly divided into a training group and a validation group (3:1). The data from the training group were used to search for independent risk factors to establish nomogram model. Data from the validation group were used to evaluate the prediction effectiveness of the nomogram model. Measurement data are expressed as mean ± standard deviation, and count data are expressed as n (%). In the training group, univariate analysis using Mann–Whitney U and Chisquared tests as appropriate was performed to assess the association between different variables and SSI. Multivariate analysis of variables with *P* < 0.1 was then performed to determine the independent risk factors for SSI [20]. Based on the regression coefficients of independent risk factors, we established a nomogram model to predict the relationship between SSI and pilon fracture.

Discrimination of dichotomous result was most often evaluated by calculating the area under the curve (AUC) of the receiver operating characteristic (ROC) curve. Generally, an AUC between 0.5 and 0.7 indicates low accuracy, 0.70–0.9 is considered acceptable, and AUC > 0.9 means that the model has excellent discriminative power [20]. ROC curves were undertaken in both the training and validation group. The calibration curve was the image comparison of predicted probabilities and actual probabilities, which was assessed using the Hosmer–Lemeshow test. Statistical analyses were carried out using EmpowerStats (http://www.empowerstats.com, X&Y Solutions, Inc., Boston, MA) and R version 4.0.2 for Windows (R Foundation for Statistical Computing, Vienna, Austria). Two-tailed analysis with P value less than 0.05 indicated that the difference was statistically significant.

## Results

From January 2012 to June 2021, 516 pilon fracture patients who underwent open reduction and internal fixation were included in this study. Of these, 387 patients were randomly assigned to the training group and 129 patients were assigned to the validation group (3:1). The baseline data of the training group and the validation group were analyzed, and there was no significant difference between the two groups (*P* > 0.05).

Table 1 showed the baseline characteristics. In the training group, 71 (18.35%) patients developed SSI with an average age of 52.1 ± 8.4 years, and 316 (81.65%) patients did not develop SSI with an average age of 47.1 ± 11.9 years (*P* < 0.001). Similar results appeared in the validation group, 23 (17.83%) patients developed SSI with an average age of 54.1 ± 11.0 years, and 106 (82.17%) patients did not develop SSI with an average age of 48.8 ± 11.2 years (*P* = 0.034). The preoperative blood sugar was significantly high in SSI patients than in non-SSI patients (7.1 ± 2.0 vs 5.9 ± 1.4, *P* < 0.001; 6.6 ± 1.8 vs 5.8 ± 1.2, *P* = 0.030; respectively). In the training and validation group, patients with prolonged operative time were more likely to develop SSI (140.0 ± 32.6 vs 100.4 ± 28.0, *P* < 0.001; 125.0 ± 29.4 vs. 87.3 ± 23.3, *P* < 0.001; respectively). Similarly, patients with multiple incisions were more likely to develop SSI (47.9% vs. 27.8%, *P* = 0.001; 60.9% vs. 31.1%, *P* = 0.007; respectively). According to results of fracture and Tscherne classification, patients with comminuted fractures and severe soft tissue injuries were more feasible to occur SSI (*P* < 0.05). There were no statistically significant differences according to gender, hemoglobin, serum albumin, C-reactive protein, blood platelet, leukocyte, waiting time for surgery, intraoperative blood loss, number of people in the operating room, cause of injury, polytrauma, drainage, bone graft, smoking or drinking.

**Table 1:** Baseline characteristics

Variable	Training group (n = 387)			Validation group (n = 129)		
	Without SSI (n = 316)	With SSI (n = 71)	*P* value*	Without SSI (n = 106)	With SSI (n = 23)	*P* value*
Age, years	47.1 ± 11.9	52.1 ± 8.4	< 0.001	48.8 ± 11.2	54.1 ± 11.0	0.034
Hemoglobin, g/L	131.3 ± 15.2	130.0 ± 16.4	0.817	129.5 ± 13.6	129.7 ± 13.1	0.887
Serum albumin, g/dL	40.4 ± 4.7	39.3 ± 5.0	0.131	40.7 ± 5.0	39.9 ± 5.8	0.671
C-reactive protein, mg/L	59.0 ± 30.3	53.6 ± 28.5	0.189	58.1 ± 26.2	55.4 ± 25.9	0.592
Blood platelet,10^∧^9/L	217.5 ± 61.9	220.9 ± 64.7	0.921	202.0 ± 66.4	219.6 ± 69.8	0.336
Leukocyte,10^∧^9/L	10.0 ± 2.9	10.6 ± 3.4	0.303	9.4 ± 2.2	9.8 ± 3.0	0.694
Preoperative blood sugar,mmol/L	5.9 ± 1.4	7.1 ± 2.0	< 0.001	5.8 ± 1.2	6.6 ± 1.8	0.030
Waiting time for surgery,days	5.4 ± 3.3	6.0 ± 4.0	0.277	5.5 ± 5.1	6.1 ± 3.3	0.107
Operative time, min	100.4 ± 28.0	140.0 ± 32.6	< 0.001	87.3 ± 23.3	125.0 ± 29.4	< 0.001
Intraoperative blood loss,ml	163.8 ± 65.4	172.0 ± 52.4	0.107	157.3 ± 53.1	170.4 ± 41.1	0.160
Number of people in the operating room	6.0 ± 1.2	6.0 ± 1.1	0.477	6.0 ± 1.2	6.3 ± 0.9	0.208
Gender			0.557			0.733
Male	190 (60.1%)	40 (56.3%)		73 (68.9%)	15 (65.2%)	
Female	126 (39.9%)	31 (43.7%)		33 (31.1%)	8 (34.8%)	
Cause of injury			0.554			0.388
Fall from height	97 (30.7%)	18 (25.3%)		22 (20.8%)	7 (30.4%)	
Traffic accident	56 (17.7%)	12 (16.9%)		28 (26.4%)	5 (21.7%)	
Hit by heavy object	32 (10.1%)	11 (15.5%)		17 (16.0%)	1 (4.4%)	
Other	131 (41.5%)	30 (42.3%)		39 (36.8%)	10 (43.5%)	
Polytrauma			0.715			0.321
No	238 (75.3%)	52 (73.2%)		77 (72.6%)	19 (82.6%)	
Yes	78 (24.7%)	19 (26.8%)		29 (27.4%)	4 (17.4%)	
Tscherne classification			0.004			0.028
Grade 0	72 (22.8%)	12 (16.9%)		23 (21.7%)	2 (8.7%)	
Grade 1	144 (45.6%)	21 (29.6%)		47 (44.3%)	6 (26.1%)	
Grade 2	94 (29.7%)	34 (47.9%)		34 (32.1%)	13 (56.5%)	
Grade 3	6 (1.9%)	4 (5.6%)		2 (1.9%)	2 (8.7%)	
Fracture classification			< 0.001			< 0.001
43.A	118 (37.4%)	9 (12.7%)		40 (37.7%)	3 (13.0%)	
43.B	141 (44.6%)	26 (36.6%)		49 (46.2%)	8 (34.8%)	
43.C	57 (18.0%)	36 (50.7%)		17 (16.1%)	12 (52.2%)	
Drainage			0.804			0.745
No	214 (67.7%)	47 (66.2%)		70 (66.0%)	16 (69.6%)	
Yes	102 (32.3%)	24 (33.8%)		36 (34.0%)	7 (30.4%)	
Bone graft			0.332			0.648
No	131 (41.5%)	25 (35.2%)		47 (44.3%)	9 (39.1%)	
Yes	185 (58.5%)	46 (64.8%)		59 (55.7%)	14 (60.9%)	
Surgical approach			0.001			0.007
Single incision	228 (72.2%)	37 (52.1%)		73 (68.9%)	9 (39.1%)	
Multiple incisions	88 (27.8%)	34 (47.9%)		33 (31.1%)	14 (60.9%)	
Smoking			0.862			0.648
No	239 (75.6%)	53 (74.6%)		83 (78.3%)	17 (73.9%)	
Yes	77 (24.4%)	18 (25.4%)		23 (21.7%)	6 (26.1%)	
Drinking			0.115			0.363
No	238 (75.3%)	47 (66.2%)		79 (74.5%)	15 (65.2%)	
Yes	78 (24.7%)	24 (33.8%)		27 (25.5%)	8 (34.8%)	

Data are presented as the mean and the standard deviation with the range in parenthesis or expressed as the number with the percentage in parenthesis. *P-value, differences between patients with pneumonia and control

In univariate analyses of the training group, the significant risk factors were age, preoperative blood sugar, Tscherne classification, fracture classification, operative time and surgical approach (*P* < 0.05). The statistically significant variables selected from the univariate analysis were included in the multivariate logistic regression analysis. Ultimately, age (OR 1.04, 95% CI 1.01–1.07), preoperative blood sugar (OR 1.66, 95% CI 1.35–2.03), operative time (OR 1.03, 95% CI 1.02–1.05), Tscherne classification (Grade 2: OR 3.97, 95% CI 1.50–10.51; Grade 3: OR 11.38, 95% CI 1.74–74.48), and fracture classification (43.C: OR 3.39, 95% CI 1.00–11.54) were identified as independent risk factors for SSI in pilon fracture patients (Table 2).

**Table 2:** Multivariable logistic regression of predictors for SSI

Variable	OR	95%CI	*P* value
Age, years	1.04	1.01, 1.07	0.022
Preoperative blood sugar,mmol/L	1.66	1.35, 2.03	< 0.001
Operative time, min	1.03	1.02, 1.05	< 0.001
Tscherne classification			
Grade 0	Ref		
Grade 1	1.22	0.46, 3.27	0.693
Grade 2	3.97	1.50, 10.51	0.006
Grade 3	11.38	1.74, 74.48	0.011
Fracture classification			
43.A	Ref		
43.B	1.76	0.66, 4.65	0.258
43.C	3.39	1.00, 11.54	0.050
Surgical approach			
Single incision	Ref		
Multiple incisions	0.81	0.37, 1.76	0.593

Data are presented as the odds ratio with the confidence interval in parenthesis. OR, odds ratio; CI, confidence interval

**Fig. 1:**
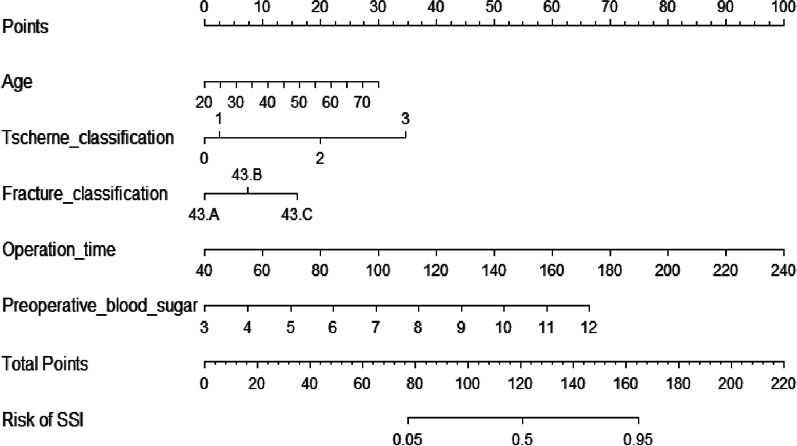
The nomogram predictive model for SSI. To use the nomogram, the points corresponding to each prediction variable were obtained, then the sum of the points was calculated as the total score, and the predicted risk corresponding to the total score was the probability of SSI

Then, we built a nomogram to predict SSI, including five independent risk factors based on multivariate logistic regression analysis (Fig. 1). Predictive model: logit(SSI) = − 12.93017 + 3.41262*I((operative time/100)^3) − 3.82443*I((operative time/100)^3 * log((operative time/100))) + 6.11450*I((preoperative blood sugar/10)^1) + 0.38065*(Tscherne classification = 2) + 1.59156*(Tscherne classification = 3) + 2.74416*(Tscherne classification = 4) + 3.79362*I((age/100)^1) + 0.23853*(fracture classification = 2) + 0.86884*(fracture classification = 3). According to the nomogram, the corresponding points of each predictor variable were obtained, the sum of the points was calculated as the total score, and the predicted risk corresponding to the total score was the probability of SSI.

**Fig. 2:**
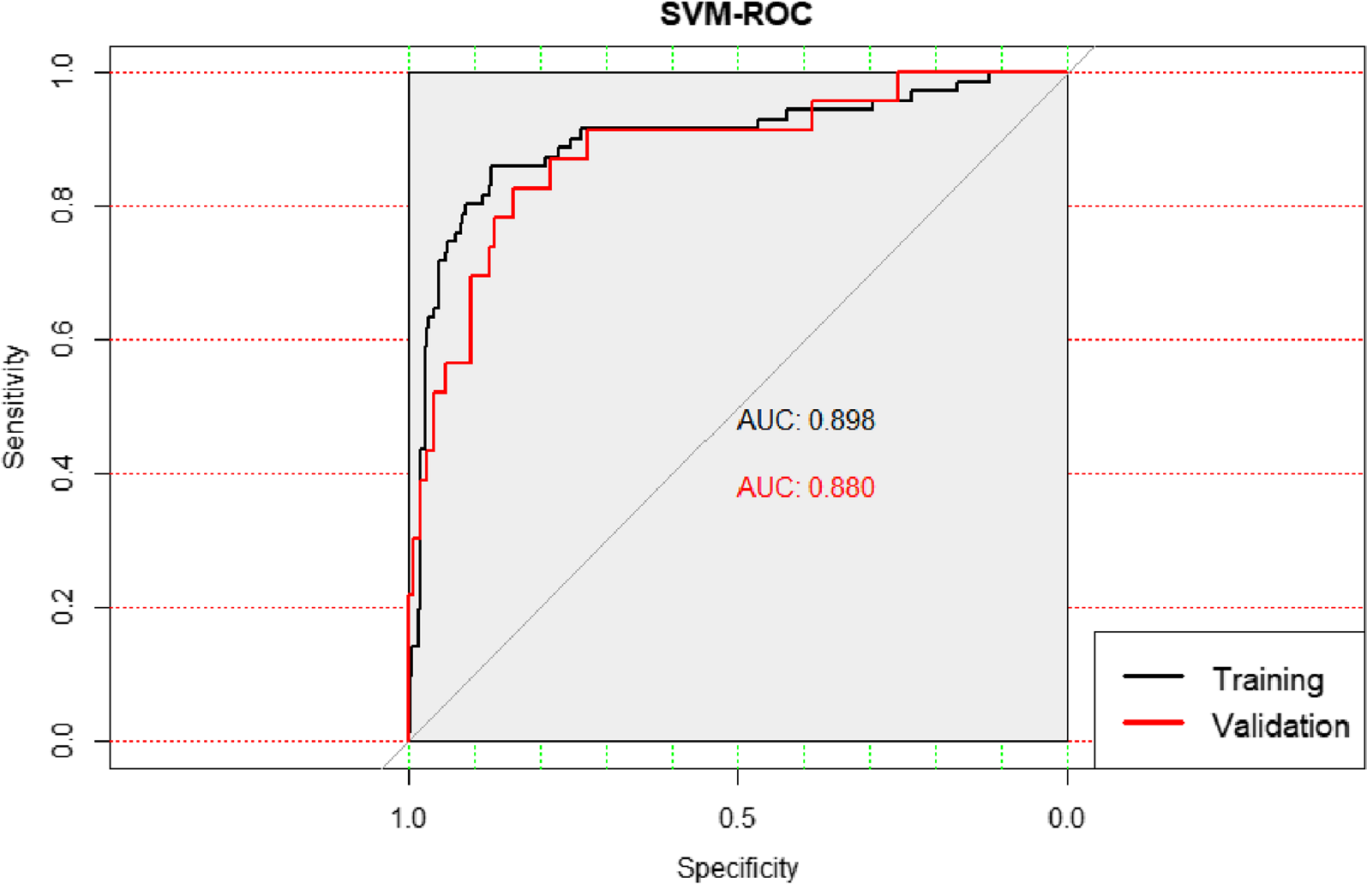
ROC curves for validating the discrimination of the nomogram predictive model (training group AUC = 0.898, validation group AUC = 0880)

**Fig. 3:**
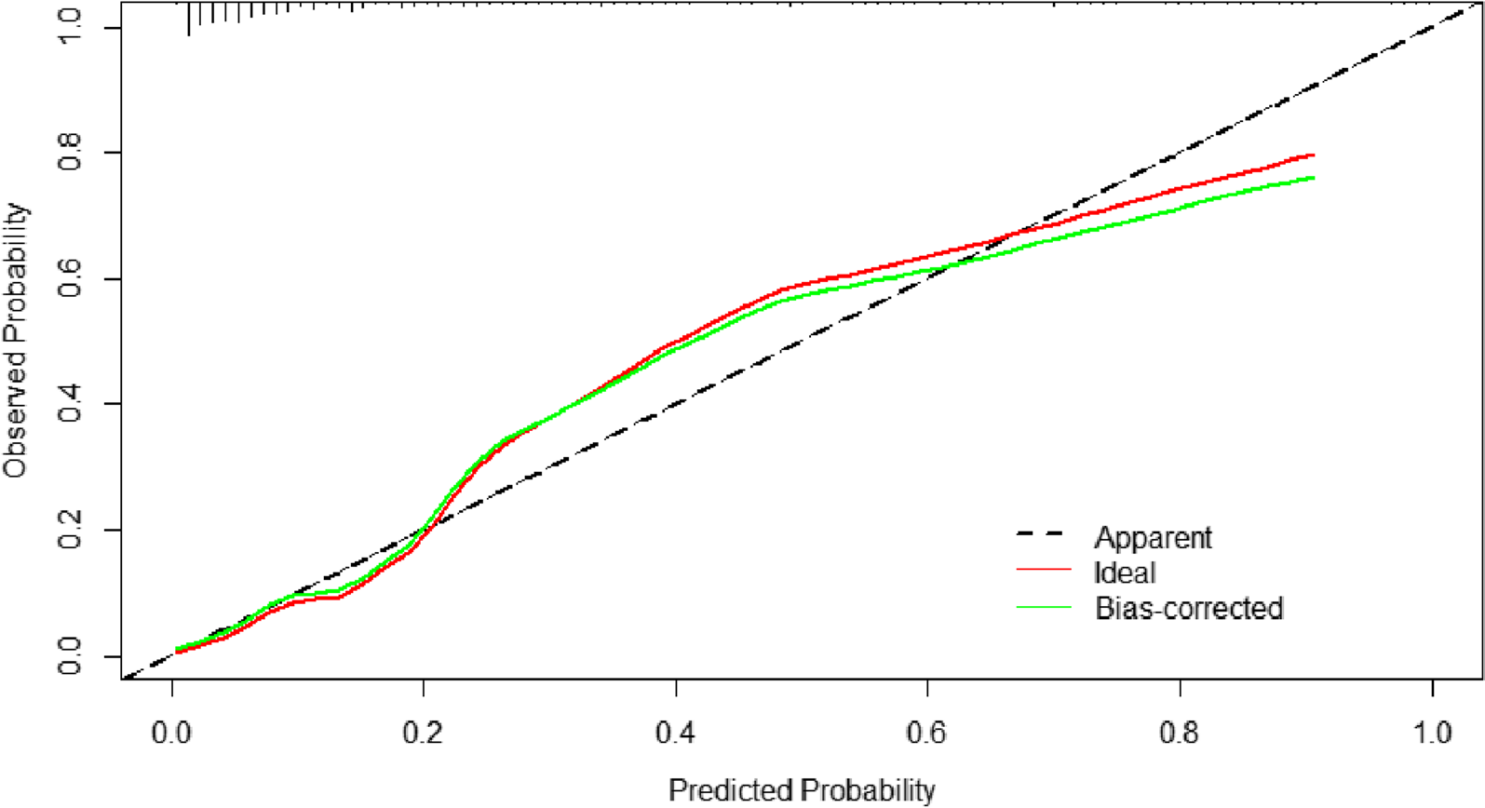
Calibration plot of the nomogram for the probability of SSI

The validation of the model was based on discrimination and calibration. We plotted the ROC curve of the predictive model and calculated the AUC value. The AUC values for SSI of the training and validation group were 0.898 and 0.880 respectively, proving that this nomogram model had good discriminative power (Fig. 2). The P value of the Hosmer–Lemeshow test was 0.125, also indicating that this nomogram model had excellent calibration ability (Fig. 3).

## Discussion

Ruedi and Allgower first published their surgical technique and early follow-up results for the treatment of pilon fractures in 1968, a key shift in treatment [21]. They proposed the principles of surgical treatment to achieve anatomical reduction and robustness of pilon fractures. First, restore the length of the fibula to reconstruct the lateral column; second, anatomically repair the articular surface of the distal tibia; third, bone graft to fill any metaphyseal bone defect, and finally place a buttress plate on the distal end of the tibia [22, 23]. However, the incidence of complications such as infection, nonunion, osteomyelitis, joint stiffness, and post-traumatic arthritis was still high. In 1999, Sirkin et al. and Patterson et al. reported a staged protocol in the treatment of severe pilon fractures, resulting in a reduced incidence of infection [24, 25].

In our research, 71 (18.35%) patients in the training group developed SSI and 23 (17.83%) patients in the validation group developed SSI. Previous studies have shown similar deep infection rates [15, 16, 26]. We found that age, preoperative blood sugar, operative time, Tscherne classification, fracture classification were considered as independent risk factors for SSI. Age is a well-known risk factor for wound healing, and older patients tend to have more comorbidities. Meng et al. and Spek et al.found that age was an independent predictor of postoperative surgical site infection in ankle fracture patients [27, 28]. A comparative study of 19,585 patients with ankle fractures showed that 30-day wound complications were significantly increased in individuals > 80 years (OR 1.84; *P* = 0.019) [29]. Results of a retrospective study of patients with OTA/AO 43C tibial pilon fractures showed that increasing age (OR 1.02, *P* = 0.040) was an independent predictor of deep infection [30].

The relationship between diabetes and SSI in pilon fractures remains unclear. Some articles reported that diabetes was not associated with deep infection in pilon fractures [30, 31]. However, other studies had shown that people with diabetes were more than twice as likely to develop deep infections [32, 33]. Our study revealed that preoperative blood glucose was an independent risk factor for SSI. Hyperglycemia can hinder wound healing and predispose patients to infections secondary to microvascular ischemia.

Operating time is a well-established risk factor for SSI and may be a marker of technical difficulties, more extensive soft tissue dissection, and prolonged wound exposure, all of which contribute to an increased incidence of SSI. Our results demonstrated that patients with prolonged operative time were more likely to develop SSI. Ren et al. believed that operative time longer than 150 min was associated with an increased risk of SSI following surgical fixation of pilon fractures [34]. It has been reported that a 15-min increase in operative time was associated with an 11% increase in risk for developing SSI [35].

Previous studies have shown that open fracture was associated with deep infection after pilon fractures, but there are few reports of closed soft tissue injuries associated with infection [12, 13, 34]. This study analyzed closed pilon fractures and found that Tscherne classification was an independent risk factor for SSI. Therefore, we believe that it is essential for soft tissue management in the perioperative period. In addition, pilon fracture type is generally considered to be associated with complications such as infection [13, 33, 34, 36]. Our results showed that the proportion of SSI in AO/OTA 43C pilon fractures was significantly higher. Complex fracture types often accompany severe soft tissue damage and also result in prolonged operative time.

To our knowledge, our article was the first study on risk factors and predictive model for SSI in closed pilon fracture patients. However, our work had some limitations. First, this study was a single-center retrospective study, and the sample size of the selected cases was relatively small. Second, the baseline characteristic data were not truly homogenous and there was bias. Third, for the validation of the predictive model we used internal data, not external data.

## Conclusion

In this study, we found that age, preoperative blood sugar, operative time, Tscherne classification and fracture classification were the independent risk factors for SSI. Our nomogram model had good discrimination and calibration power, so it could be used to predict SSI risk in patients with pilon fracture.

## Data Availability

The data used to support the findings of this study are available from the corresponding author upon request. Patient data comes from our hospital's medical records follow-up database, transparent and available.

## References

[1] Michelson J, Moskovitz P, Labropoulos P (2004). The nomenclature for intra-articular vertical impact fractures of the tibial plafond: pilon versus pylon. Foot Ankle Int.

[2] Mauffrey C, Vasario G, Battiston B, Lewis C, Beazley J, Seligson D (2011). Tibial pilon fractures: a review of incidence, diagnosis, treatment, and complications. Acta Orthop Belg.

[3] Luo TD, Eady JM, Aneja A, Miller AN (2017). Classifications in brief: Rüedi-Allgöwer classification of Tibial plafond fractures. Clin Orthop Relat Res.

[4] Chen H, Cui X, Ma B, Rui Y, Li H (2019). Staged procedure protocol based on the four-column concept in the treatment of AO/OTA type 43-C3.3 pilon fractures. J Int Med Res.

[5] Krettek C, Bachmann S (2015). Pilon fractures. Part 1: Diagnostics, treatment strategies and approaches. Chirurg.

[6] Saad BN, Yingling JM, Liporace FA, Yoon RS (2019). Pilon fractures: challenges and solutions. Orthop Res Rev.

[7] Müller M, Nazarian S, Koch P, Schatzker J (1990). The comprehensive classification of fractures of long bones.

[8] Ibrahim DA, Swenson A, Sassoon A, Fernando ND (2017). Classifications in brief: the tscherne classification of soft tissue injury. Clin Orthop Relat Res.

[9] Kottmeier SA, Madison RD, Divaris N (2018). Pilon fracture: preventing complications. J Am Acad Orthop Surg.

[10] Rubio-Suarez JC, Carbonell-Escobar R, Rodriguez-Merchan EC (2018). Fractures of the tibial pilon treated by open reduction and internal fixation (locking compression plate-less invasive stabilising system): complications and sequelae. Injury.

[11] Chen Y, Huang XY, Chen YL, Shi C, Li H, Xu J (2021). Comparison of complications of early and delayed open reduction and internal fixation for treating pilon fracture: a protocol of systematic review and meta-analysis. PLoS ONE.

[12] Lu V, Zhang J, Zhou A, Thahir A, Lim JA, Krkovic M (2022). Open versus closed pilon fractures: comparison of management, outcomes, and complications. Injury.

[13] Molina CS, Stinner DJ, Fras AR, Evans JM (2015). Course of treatment and rate of successful salvage following the diagnosis of deep infection in patients treated for pilon fractures (AO/OTA: 43). J Orthop.

[14] Joveniaux P, Ohl X, Harisboure A, Berrichi A, Labatut L, Simon P (2010). Distal tibia fractures: management and complications of 101 cases. Int Orthop.

[15] Potter JM, Vliet QMJ, Esposito JG, McTague MF, Weaver M, Heng M (2019). Is the proximity of external fixator pins to eventual definitive fixation implants related to the risk of deep infection in the staged management of tibial pilon fractures?. Injury.

[16] Esposito JG, Vliet QMJ, Heng M, Potter J, Cronin PK, Harris MB (2020). Does surgical approach influence the risk of postoperative infection after surgical treatment of Tibial pilon fractures?. J Orthop Trauma.

[17] Miller AG, Margules A, Raikin SM (2012). Risk factors for wound complications after ankle fracture surgery. J Bone Joint Surg Am.

[18] Thangarajah T, Prasad PS, Narayan B (2009). Surgical site infections following open reduction and internal fixation of ankle fractures. Open Orthop J.

[19] Berríos-Torres SI, Umscheid CA, Bratzler DW, Leas B, Stone EC, Kelz RR, et al; Healthcare Infection Control Practices Advisory Committee. Centers for Disease Control and Prevention Guideline for the Prevention of Surgical Site Infection, 2017. JAMA Surg. 2017;152:784–91. 10.1001/jamasurg.2017.0904. 10.1001/jamasurg.2017.0904 28467526

[20] Xiang G, Dong X, Lin S, Cai L, Zhou F, Luo P (2023). A nomogram for prediction of deep venous thrombosis risk in elderly femoral intertrochanteric fracture patients: a dual-center retrospective study. Front Surg.

[21] Rüedi T, Matter P, Allgöwer M (1968). Intra-articular fractures of the distal tibial end. Helv Chir Acta.

[22] Rüedi T (1973). Fractures of the lower end of the tibia into the ankle joint: results 9 years after open reduction and internal fixation. Injury.

[23] Rüedi TP, Allgöwer M (1979). The operative treatment of intra-articular fractures of the lower end of the tibia. Clin Orthop Relat Res.

[24] Sirkin M, Sanders R, DiPasquale T, Herscovici D (1999). A staged protocol for soft tissue management in the treatment of complex pilon fractures. J Orthop Trauma.

[25] Patterson MJ, Cole JD (1999). Two-staged delayed open reduction and internal fixation of severe pilon fractures. J Orthop Trauma.

[26] Dombrowsky A, Abyar E, McGwin G, Johnson M (2021). Is definitive plate fixation overlap with external fixator pin sites a risk factor for infection in Pilon fractures?. J Orthop Trauma.

[27] Meng J, Sun T, Zhang F, Qin S, Li Y, Zhao H (2018). Deep surgical site infection after ankle fractures treated by open reduction and internal fixation in adults: A retrospective case-control study. Int Wound J.

[28] Spek RWA, Smeeing DPJ, van den Heuvel L, Kokke MC, Bhashyam AR, Kelder JC (2021). Complications after surgical treatment of geriatric ankle fractures. J Foot Ankle Surg.

[29] Gray MT, Hidden KA, Malik AT, Khan SN, Phieffer L, Ly TV (2021). Octogenarian and nonagenarians are at a higher risk for experiencing adverse 30-day outcomes following ORIF of ankle fractures. Geriatr Orthop Surg Rehabil.

[30] Olson JJ, Anand K, von Keudell A, Esposito JG, Rodriguez EK, Smith RM (2021). Judicious use of early fixation of closed, complete articular pilon fractures is not associated with an increased risk of deep infection or wound complications. J Orthop Trauma.

[31] Spitler CA, Hulick RM, Weldy J, Howell K, Bergin PF, Graves ML (2020). What are the risk factors for deep infection in OTA/AO 43C pilon fractures?. J Orthop Trauma.

[32] Kline AJ, Gruen GS, Pape HC, Tarkin IS, Irrgang JJ, Wukich DK (2009). Early complications following the operative treatment of pilon fractures with and without diabetes. Foot Ankle Int.

[33] Yeramosu T, Satpathy J, Perdue PW, Toney CB, Torbert JT, Cinats DJ (2022). Risk factors for infection and subsequent adverse clinical results in the setting of operatively treated pilon fractures. J Orthop Trauma.

[34] Ren T, Ding L, Xue F, He Z, Xiao H (2015). Risk factors for surgical site infection of pilon fractures. Clinics (Sao Paulo).

[35] Gowd AK, Bohl DD, Hamid KS, Lee S, Holmes GB, Lin J (2020). Longer operative time is independently associated with surgical site infection and wound dehiscence following open reduction and internal fixation of the ankle. Foot Ankle Spec.

[36] Kilian O, Bündner MS, Horas U, Heiss C, Schnettler R (2002). Long-term results in the surgical treatment of pilon tibial fractures. A retrospective study. Chirurg.

